# Correction: White et al. AFOs Improve Stride Length and Gait Velocity but Not Motor Function for Most with Mild Cerebral Palsy. *Sensors* 2023, *23*, 569

**DOI:** 10.3390/s25226862

**Published:** 2025-11-10

**Authors:** Hank White, Brian Barney, Sam Augsburger, Eric Miller, Henry Iwinski

**Affiliations:** 1 Shriners Children’s Lexington, 110 Conn Terrace, Lexington, KY 40508, USA; 2 Division of Physical Therapy, College of Health Sciences, University of Kentucky, 900 South Limestone, Lexington, KY 40536, USA; 3 Pediatric Orthotic & Prosthetic Services—Midwest, 110 Conn Terrace, Lexington, KY 40508, USA; 4 Department of Orthopaedic Surgery, University of Kentucky, 740 S Limestone St., Room K423, Lexington, KY 40536, USA

The Editorial Office and Editorial Board of *Sensors* are jointly issuing a resolution and removal of the *Journal Notice* linked to this article [1], as well as an update to the original publication. Following concerns raised about the integrity of the peer review, the Editorial Office has conducted a post-publication peer review of this article. This process included the recruitment of a new independent reviewer and was supervised by the Section Editor-in-Chief to ensure full compliance with MDPI’s Editorial Process (https://www.mdpi.com/editorial_process (accessed on 10 October 2025)).

As a result of this process, the Section Editor-in-Chief and the authors have agreed to update the following aspects of this publication:

- Reviewer report 1 was removed from the peer review record and Reviewer report 3 was uploaded to the peer review record (https://www.mdpi.com/1424-8220/23/2/569/review_report (accessed on 10 October 2025)).
- The original Academic Editors listed on this publication were removed and replaced with the Section Editor-in-Chief, who conducted this post-publication review.

With this update, the Academic Editor is satisfied that the Editorial Process relating to this article was completed as per MDPI’s Editorial Process policy. The Editorial Office would like to thank the authors for their collaboration during this process. 

The Editorial Office confirms that this change does not affect the scientific results.

The original publication has also been updated.
